# It is the time for quorum sensing inhibition as alternative strategy of antimicrobial therapy

**DOI:** 10.1186/s12964-023-01154-9

**Published:** 2023-06-14

**Authors:** Nourhan G. Naga, Dalia E. El-Badan, Khaled M. Ghanem, Mona I. Shaaban

**Affiliations:** 1 Botany and Microbiology Department, Faculty of Science, Alexandria University, Alexandria, Egypt; 2 Department of Biological Sciences, Faculty of Science, Beirut Arab University, Beirut, Lebanon; 3 Microbiology and Immunology Department, Faculty of Pharmacy, Mansoura University, Mansoura, Egypt

**Keywords:** Virulence factors, Antibiotics, Quorum sensing, Autoinducers, Multiple drug resistance

## Abstract

Multiple drug resistance poses a significant threat to public health worldwide, with a substantial increase in morbidity and mortality rates. Consequently, searching for novel strategies to control microbial pathogenicity is necessary. With the aid of auto-inducers (AIs), quorum sensing (QS) regulates bacterial virulence factors through cell-to-cell signaling networks. AIs are small signaling molecules produced during the stationary phase. When bacterial cultures reach a certain level of growth, these molecules regulate the expression of the bound genes by acting as mirrors that reflect the inoculum density.

Gram-positive bacteria use the peptide derivatives of these signaling molecules, whereas Gram-negative bacteria use the fatty acid derivatives, and the majority of bacteria can use both types to modulate the expression of the target gene. Numerous natural and synthetic QS inhibitors (QSIs) have been developed to reduce microbial pathogenesis. Applications of QSI are vital to human health, as well as fisheries and aquaculture, agriculture, and water treatment.

Video Abstract

**Supplementary Information:**

The online version contains supplementary material available at 10.1186/s12964-023-01154-9.

## Introduction

The discovery of antibiotics by Sir Alexander Fleming in 1945 put an end to infectious diseases. However, in recent decades, the efficacy of these extraordinary drugs has diminished. Microorganisms must adapt to survive, which could be accomplished by the evolution of antimicrobial resistance. The development of resistance by microorganisms was not surprising, as it is a typical survival mechanism. According to the World Health Organization (WHO), multiple drug resistance (MDR) is one of the top ten severe threats to public health, with high morbidity and mortality rates worldwide. The emergence of antibiotic resistance in pathogenic Gram-positive and Gram-negative bacteria, such as methicillin-resistant *Staphylococcus aureus* (MRSA), vancomycin-resistant *Staphylococcus aureus* (VRSA), vancomycin-resistant *Enterococci* (VRE), and carbapenem-resistant *Klebsiella pneumoniae* (CRKP) poses a threat to humans. The world has experienced seven epidemics caused by infectious organisms, with *Vibrio cholerae* ranking first [1]. *Pseudomonas aeruginosa*,* S. aureus*, *Escherichia coli*, and *Candida albicans* are the leading causes of the increasing number of deaths in hospitals and intensive care units [2].

MDR is a complicated issue that has recently been exacerbated by multiple factors. Continuous exposure to some antibiotics certain antibiotics has enabled the development of multidrug and extensive drug resistance [3]. In Gram-negative bacteria, plasmid-mediated resistance to quinolones has been reported [4]. Due to the production of the β-lactamase enzyme, other bacteria can develop resistance to most β-lactam antibiotics. In addition, MDR can also be acquired through genetic mutation and horizontal gene transfer [5].

**Fig. 1:**
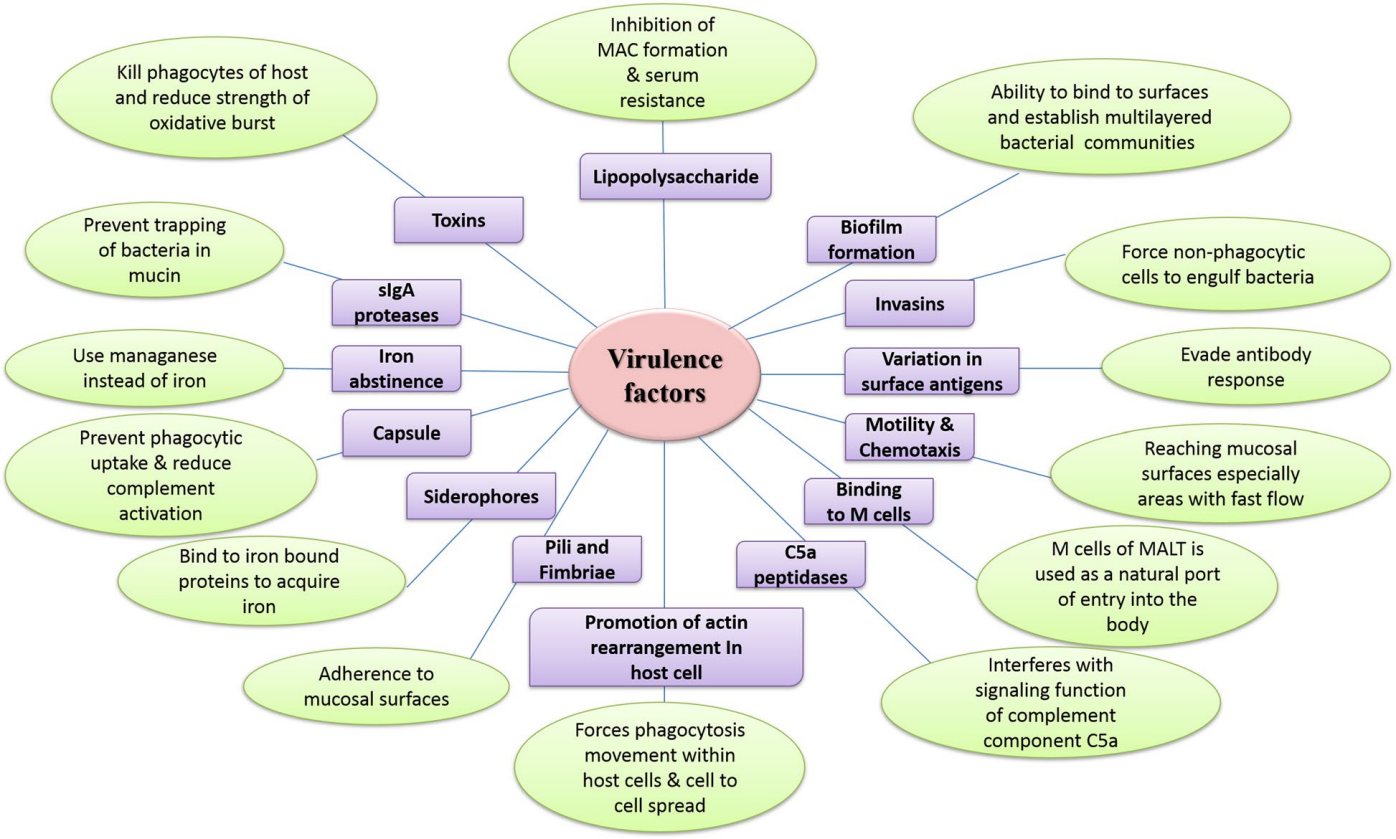
Various virulence factors of pathogenic bacteria regulated by quorum sensing

Furthermore, social factors, such as the absence of government policies and the self-medication of consumers who are not certified health professionals, have contributed to the development of MDR. For instance, antimicrobial drugs can be purchased over the counter in developing nations. Lack of medical knowledge has led to inappropriate prescriptions, such as prescribing antibiotics for influenza and inadequate doses. The misuse and overuse of antibiotics diminished their efficacy against infectious diseases and contributed to the emergence of microbial resistance [6]. External factors, such as the inappropriate use of antibiotics in poultry, aquaculture, and agriculture, have contributed to MDR. Infection with drug-resistant bacteria is now significantly more prevalent than infection with drug-susceptible organisms. Antibiotics are released into the environment through domestic use, hospitals, pharmaceutical companies, and research facilities contaminating drinking water similarly to other drugs. The Food and Drug Administration (FDA) and the Environmental Protection Agency (EPA) have not yet developed legislation and regulations, resulting in a significant problem for all forms of life, including aquatic ecosystems [7]. The spread of MDR has challenged researchers to develop new methods in order to overcome this problem. One of these methods is to control bacteria’s virulence and pathogenic factors. Many virulence factors are controlled by cell-to-cell signaling pathways communicative network known as QS [8] (Fig. 1). QS is a ubiquitous phenomenon in the bacterial world that plays a significant role in controlling myriad activities and facilitating survival in hostile environments [9]. For instance, it causes some changes in gene expression resulting in phenotypic modulation in bacteria [10]. As a result, processes like biofilm formation, development of genetic competence, transfer of conjugative plasmid, regulation of virulence, sporulation, symbiosis, and the production of antimicrobial peptides contribute to bacterial adaptation to stress during growth and harsh environmental conditions [11] and antibiotic action [12].

### Auto-inducers

**Fig. 2:**
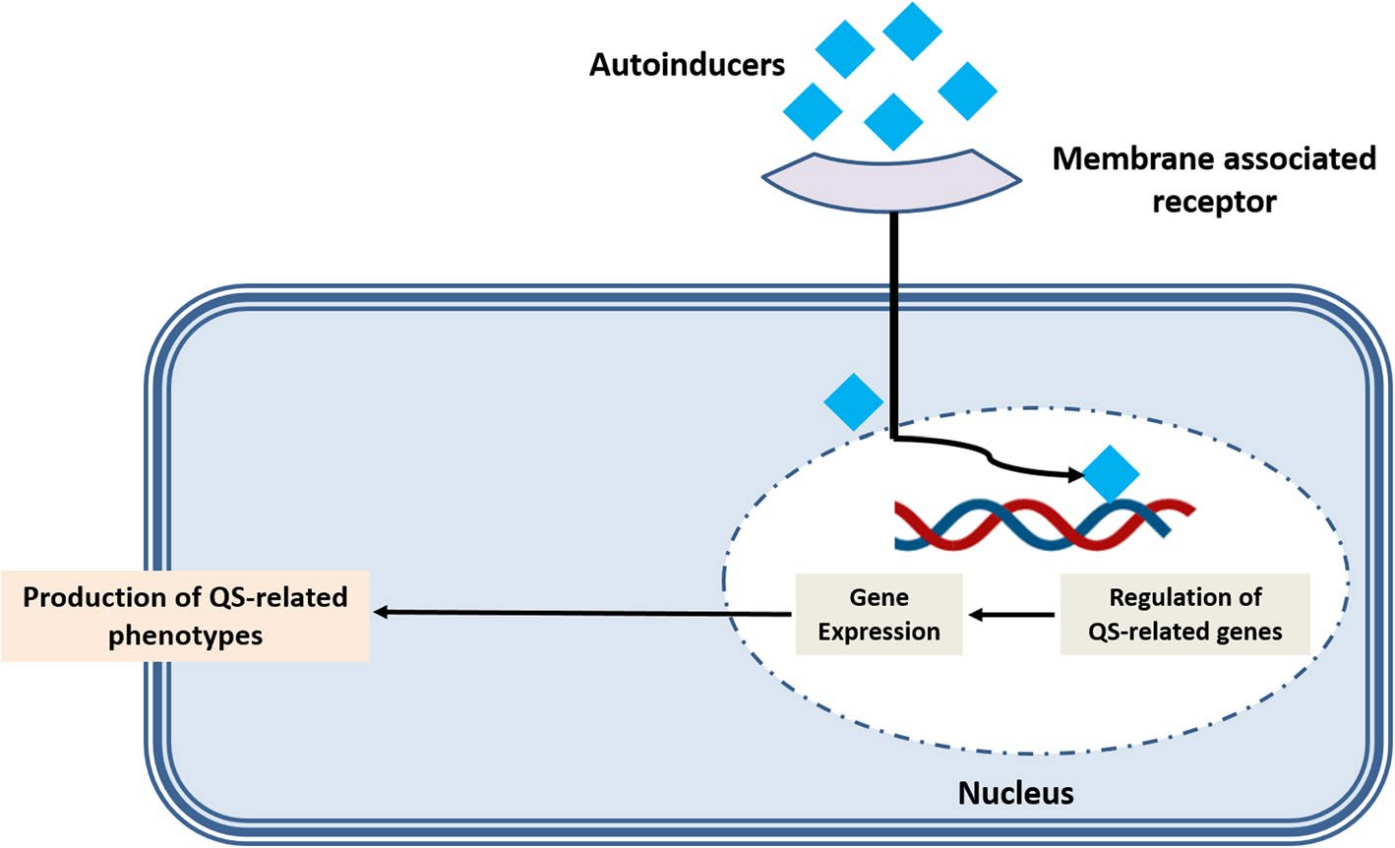
Mechanism of action of auto-inducers

QS promotes communication among bacterial cells by sensing and releasing AIs. AIs are small signaling molecules produced in the stationary phase at the basal level [13]. These molecules act as mirrors that reflect the density of the inoculum. Once the threshold of the growth is reached, they regulate the expression of related genes [14, 15] (Fig. 2). Gram-positive bacteria use peptide derivatives as signaling molecules, whereas Gram-negative bacteria use fatty acid derivatives. Most bacteria can use both AI types of AIs to modulate the target gene expression [16].

### Classes of auto-inducers

#### Acyl homoserine lactone

**Fig. 3:**
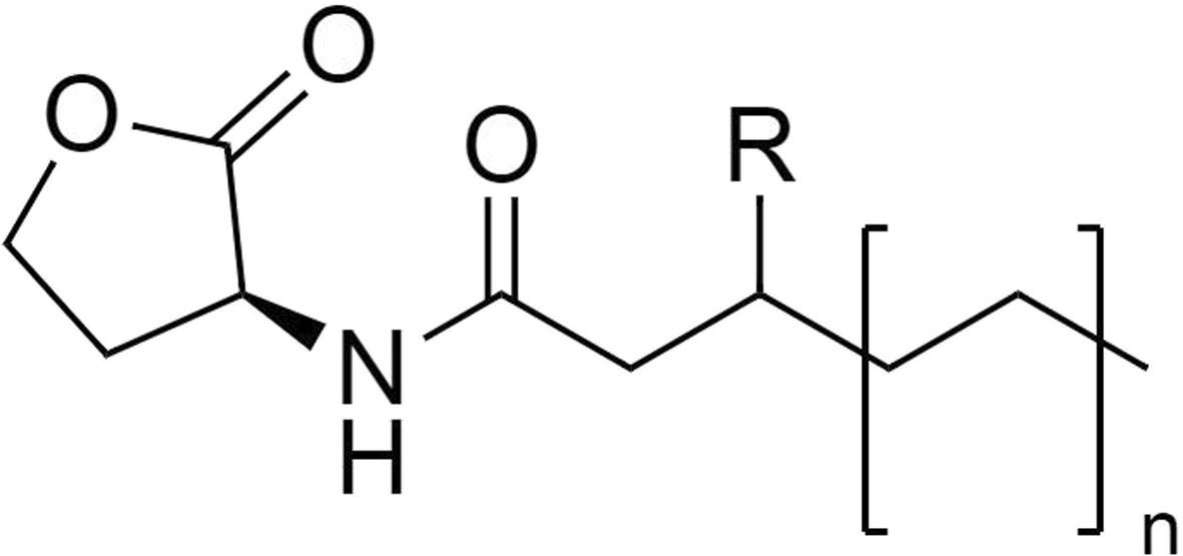
Acyl homoserine lactone

Acyl homoserine lactone (AHL) consists of a lactone ring and a side chain of carbon atoms ranging from C8 to C14 in length (Fig. 3). They occur primarily in Gram-negative bacteria and are involved in intraspecies communication. Moreover, they are produced by a group of homologous LuxI (AHL synthase) proteins that use S-adenosyl methionine as a building block, providing the homoserine lactone moiety. At low cell density, low concentrations of LuxI are produced, followed by the production of AHLs at low concentrations that can diffuse freely through the cell membrane. AHLs accumulate with the bacterial growth until the threshold level at which the transcriptional activating protein LuxR (AHL receptor) binds to the AHL molecules [16]. The AHL-LuxR complex forms dimers or multimers then binds to its cognate promotor and activates QS-related bacterial gene expression [14, 17–19]. Most AHL biosensors have a variety of similar topologies for detecting QS gene networks: (a) a QS transcription activator expressed by an induced or constitutive promoter: and (b) a reporter gene expressed by the LuxR’s cognate promoter homolog.

### Auto-inducing peptides

As QS molecules, Gram-positive bacteria produce auto-inducing peptides (AIPs). AIPs are small, heterogeneous oligopeptides with a linear or cyclic structure [20]. At high doses, AIP binds to histidine kinase (HK) receptors. The autophosphorylation of the HK receptor activates the cytoplasmic regulator, which then stimulates the transcription of the QS-related genes. After being released from the HK receptor, the AIPs are recirculated within the cell cytoplasm, where they regulate the activity of transcription factors [17].

### Auto-inducers-2

Auto-inducers-2 (AI-2) consist of furanosyl borate diester and 4, 5-dihydroxy- 2,3-pentane dione (DPD) derivatives. It is found in Gram-negative and Gram-positive bacteria and is produced by intraspecies. In addition, it is regarded as the most prevalent signaling molecule [18]. The mechanism of action of AI-2 is still uknown, but it occurs by activation of the Lsr transport system by the phosphoenolpyruvate phosphotransferase system [21]. AI-2 was originally identified in the bioluminescence system of the marine bacterium *Vibrio harveyi* and is synthesized by two complex components, one of which is catalyzed by luxS gene locus and related homologs and the other is catalyzed by the S-adenosyl homocysteine nucleosidase enzyme [17]. AI-2 regulates different activities in many bacterial species, including biofilm formation in *V. cholerae*, *Streptococcus mutans*, and *Salmonella Typhimurium* [22]. Moreover, they modulate motility in *E. coli* and* Campylobacter jejuni* [23, 24]. The conjugation of AI-2 with AIPs and AHLs can regulate a wide variety of bacterial properties, such as the growth of *Bacillus anthracis*, pathogenicity of *V. cholerae*, and bioluminescence of *V. harvey*i [25].

### Methods to screen AI producers

QS molecules can regulate an excessive number of physiological processes that directly affect human and plant life, so it is crucial to study the properties of these ubiquitous small molecules. Quantitative and qualitative methods to characterize and detect AIs have been developed recently. The optimal method is determined by the nature of the study and the type of autoinducer manufactured [26]

### T-Streak plate method

Auto-inducer molecules can be detected by QS biosensor strains, and recently, numerous biosensors have been developed to detect them and detect either AI-1 or AI-2. Both detecting the presence of AIs in bacteria and screening for QS inhibitors are effective [27]. The bacterial biosensor is a genetically modified bacterium with a remarkable QS gene circuit and a reporter gene circuit that is simple to measure and detect. The reporter gene generates a variety of observable outputs, including luminescence, fluorescence, and color pigments. It is the most commonly used technique due to being fast, inexpensive, and easy to apply for qualitatively screening AIs [26]. The tester strain is streaked on agar, producing a distinguished visible output such as *Chromobacterium violaceum,* the large motile Gram-negative cosmopolitan β-proteobacteria that produce a violet pigment termed “violacein” [28].

*C. violaceum* is the most frequently used biosensor. Its QS signaling system utilizes N-hexanoyl and N-decanoyl-L-homoserine lactone mediated by CviI and CviR (LuxI/LuxR) homolog genes. CviI AHL-synthase is responsible for C10-HSL synthesis, and CviR is the transcriptional regulatory protein that binds to AHL molecules on reaching the threshold level. This results in the formation of a complex that activates *C. violaceum* QS-regulated genes such as violacein pigment production, biofilm formation, chitinase, lipase, exopolysaccharide (EPS), and flagellar proteins [29–32]. Other types of reporter strains, such as *E. coli* JM109 ( [33], *S. aureus* 8325–4 [34], and *Acinetobacter baumannii* ATCC 19,606 and ATCC 17,978 can be utilized [35].

### Thin-Layer Chromatography

The thin-layer chromatography (TLC) method is more accurate and sensitive than the streak plate method because it provides information on both the type and size of AIs in the tester strain. AIs are organic compounds that must be separated using organic solvents [26]. The supernatants are placed on a TLC plate, dried to allow separation, and then covered with agarose containing the biosensor strain. On the TLC plate, a tear or circular shape is created for a specific type of visible spot when a biosensor and AI are combined. This shape can predict the presence of a specific AI in the tester strain, but mass spectrometry is required for confirmation [36].

### Calorimetric assay

The calorimetric assay is an accurate method that is used for quantitative and qualitative detections of AHLs. However, it does not provide any information regarding the size or type of AHLs. The biosensor should be grown with a test strain and O-nitrophenyl-beta-D-galactopyranoside (ONPG). The galactosidase enzyme is produced as a result of converting ONPG to ortho-nitrophenol and galactose. It is a consistent method for biosensors that use the lacZ reporter gene. In this case, ortho-nitrophenol can be quantified by the Miller test method using a spectrophotometer at 420 nm. The quantity of AHL in the test strain may also be helpful in determining the AHL standard curve [26, 37].

### Luminescence assay

The luminescence test provides a qualitative determination of AIs, and it can also give a quantitative determination by drawing a standard curve. This test is carried out using liquid test strain extracts mixed with a biosensor strain. This method is ideal, especially for biosensors that utilize luxCDABE reporter luminescence genes, and is comparable to test [38]. In addition, it is highly accurate but provides no information regarding the nature or size of the AHL. It is detected using a luminometer, and the assay is performed on an extract of the test and biosensor strains [39]**.**

### Strategies for disrupting the signaling systems

There are at least two significant areas of research that focus on finding antibiotic alternatives. The first is antimicrobial peptide application (AMP), which involves the use of small, effective molecules against resistant pathogens [40]. The second approach involves searching for natural and synthetic substances with QSI activity that targets cell–cell communication and thus control pathogenesis [41].

### Targeting the biosynthesis of AIs

Minimizing cell-to-cell communication and hindering their formation plays a vital role in QS inhibition and limits the emergence of pathogenic symptoms. This strategy inhibits the production of AHLs and AI-2, which are responsible for interspecies and intraspecies communication. This method is rarely employed for deactivation and signal degradation.

### Blocking of AHLs production in Gram-Negative Bacteria

**Fig. 4:**
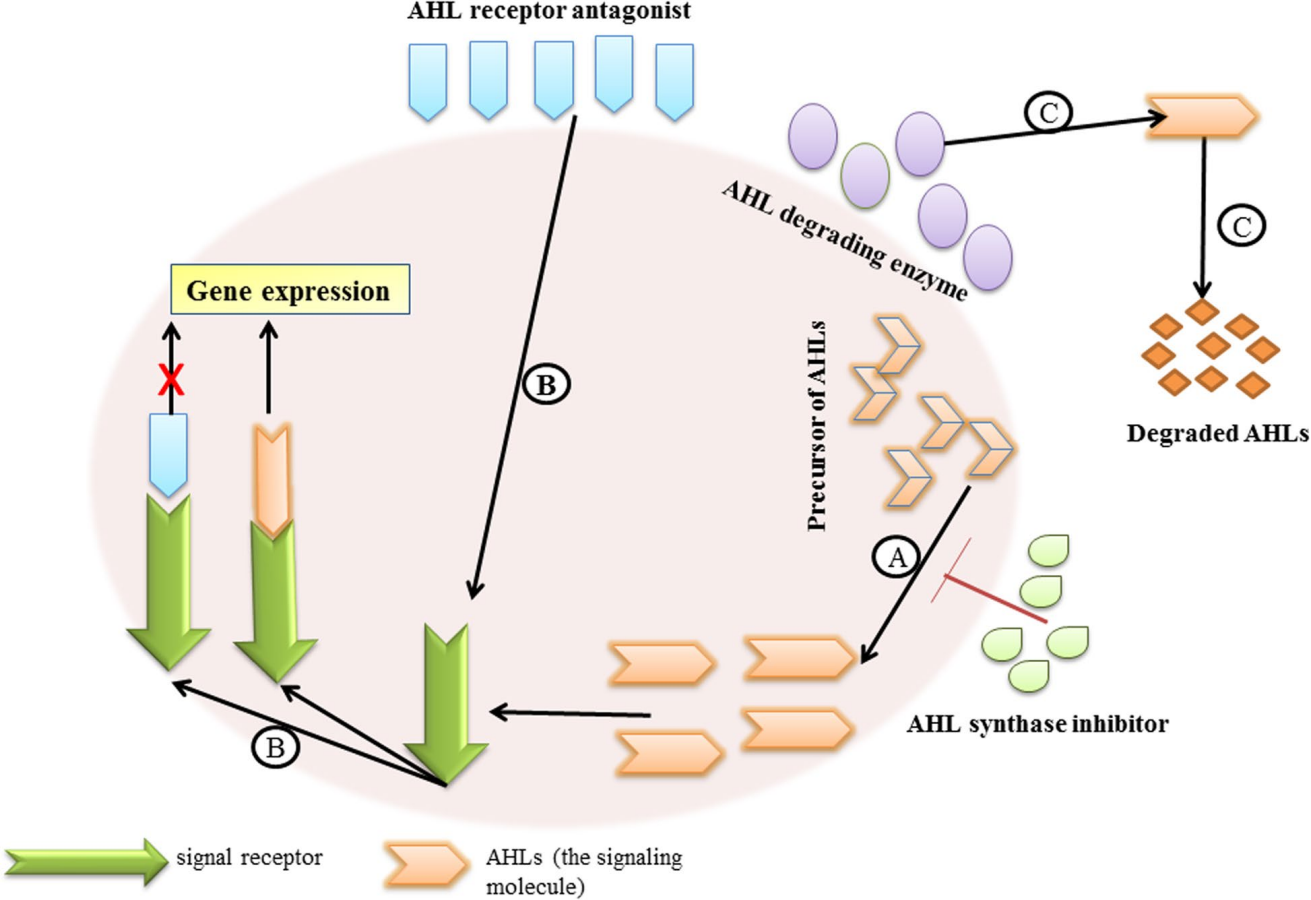
Mechanisms of quorum sensing inhibition

The three enzymatic systems that produce AHL synthase enzyme are HdtS, LuxI, and LuxM; however, the most frequent target of these enzymes is Lux I-type synthase [42] (Fig. 4A). The most studied AHL synthase inhibitors are structural analogs of S-adenosylmethionine (SAM). It has been reported that compounds such as sinefungin, L/D-S-adenosyl homocysteine, and butyryl-SAM inhibited the first step in QS signaling and suppressed AHL synthesis in pathogenic *P. aeruginosa* in vitro [43]. Notably, SAM is a fundamental component of other enzymes in biological systems, and its analogs may inhibit AHL production [44, 45]. For instance, when *E. coli* was cultivated in a methionine-free environment, all elements of the bacterial methyl cycle were reduced [46]. The lack of methyl cycle components affects the production of the AHL signal because it is composed of carbon, which is derived from the fatty acid biosynthesis intermediates of the host [47].

### Blocking of AIPs production in Gram-positive bacteria

There is a paucity of studies on blocking AIPs because AIPs are synthesized by ribosomes, and peptidase enzymes are also included in bacteria growth and survival. Consequently, their inhibition is bactericidal and contributes to the emergence of bacterial resistance [41]. Nevertheless, numerous studies have been conducted to comprehend the mechanism of signal mechanisms, which will eventually lead to the development of signal inhibition methods[45, 48].

### Targeting the AI-2 synthases

Targeting the LuxS enzyme splits S-ribosyl-L-homocysteine (SHR) to produce 4,5 dihydroxy-2,3- pentanedione (DPD) and L-homocysteine could disrupt AI-2 and mitigate microbial pathogenesis. S-homoribosyl-L-cysteine and L-homocysteine were approved because they could inhibit LuxS synthases and SHR hydrolysis across a wide spectrum of bacteria [49]. n addition, numerous naturally occurring brominated furanone have been demonstrated to inhibit the LuxS enzyme in a concentration-dependent manner [50]. Numerous combinations of brominated furanone have been synthesized and evaluated for their ability to inhibit QS processes in the *V. harveyi* reporter strain [51]. *V. harveyi* can only respond to cross-bacterial communication using the AI-2 signal because it does not have the AHL signal receptor. The presence of furanone derivatives has been shown to reduce the bioluminescence in *V. harveyi*, indicating their potential role in disrupting bacterial communication. Additionally, furanone reduced the biofilm formation of *S. epidermidis* by 57% [51]. Natural compounds like surfactin that have been isolated from *Bacillus subtilis* have the potential to target the LuxS/AI-2 QS system and inhibit biofilm formation by 70% at 1/2 MIC (16 μg/mL) [52].

### Targeting AI receptors

Numerous quorum sensing inhibitors (QSIs) inhibit bacterial signal receptors, resulting in an inactive receptor–signal complex that inhibits cell-to-cell communication and limits the pathogenesis and virulence of infectious bacteria. Targeting AI signal receptors is the most obvious strategy, but not all of the listed QSIs have been identified as AI receptor-disrupting agents [44].

### Targeting receptors of AHL in Gram-negative bacteria

LuxR-AHL is the most widespread IA receptor protein found in Gram-negative bacteria. Consequently, the targeting of this complex by certain QSIs is an effective alternative strategy for controlling pathogenesis (Fig. 4B). Strategies to disrupt the bond between the signal receptor protein and AHLs are based on the synthesis and design of inhibitors like AHL analogs, structurally independent AHLs, and naturally occurring QS inhibitors.

### AHL analogs

Cell-to-cell communication via AHL signals is primarily determined by their chemical structure (lactone ring and acyl side chain). Since any change in their structure, such as the incorporation of any functional group in the acyl side chain, changes in chirality and geometry block the interaction between the receptor and signal. For instance, when the active methylene group was incorporated into the AHL, the protein-signal binding of the receptor decreased by 50%, and when an additional methylene group was added, the activity was reduced by 90%. Therefore, the AHL modifications represent a highly effective strategy for controlling processes with QS signals [33]. Several bulky groups that could inhibit the LasR, TraR, and LuxR receptors have been added to the acyl side chain to create certain AHL analogs in *P. aeruginosa*, *Agrobacterium tumefaciens*, and *Vibrio fischeri*, respectively [53].

### Structural unrelated AHLs

Although a 90% loss of binding capacity was achieved in-vitro, actual application in-vivo is still very limited, and more structurally unrelated AHL signals as alternative compounds need to be discovered. It has been demonstrated that certain antibiotics in sub-minimum inhibitory concentration (MIC) doses, such as ciprofloxacin, ceftazidime, azithromycin, cefoperazone, and various synthetic furanone, can reduce virulence factors like motility and biofilm formation in some Gram-negative bacteria and inhibit QS signaling [54]. In addition, the efficacy of nonsteroidal anti-inflammatory drugs (NSAIDs) such as piroxicam and meloxicam was approved for efficacy as QSIs [55]. Similarly, nanoparticles (NPs) could attenuate the pathogenesis of some pathogenic bacteria. For instance, copper ion NPs inhibited the Qs signaling system of *P. aeruginosa* [56], gold NPs in *V.cholerae* [57]*,* and silver NPs against *P. aeruginosa* [58]*.*

### Natural QS inhibitors analogs

Several natural compounds have the potential to inhibit the QS signaling systems of certain pathogens. For example, the limonene compound extracted from *Citrus reticulate* inhibited biofilm formation by 41% at 0.1 mg/mL and AHL signaling production by 33% in *Pseudomonas aeruginosa* [59]. Moreover, the same compound was isolated from *Eucalyptus radiate* and could inhibit QS-regulated pyomelanin pigment production in *A. baumannii* [60]. Similarly, the phenolic extract of *Rubus rosifolius* inhibited swarming motility and biofilm formation in *Serratia marcescens* [61].

Pyocyanin production, biofilm formation, swarming motility, elastolytic, and proteolytic activities in *P. aeruginosa* PAO1 were inhibited in a concentration-dependent manner by a flavonoid-rich fraction of *Centella Asiatica* [62]*.* The methoxyisoflavan compound isolated from *Trigonella stellate* reduced violacein production in *C. violaceum* and inhibited pyocyanin, protease production, hemolysin activity, and biofilm formation in *P. aeruginosa* [63]. The biofilm formation of *Escherichia coli* and *Pseudomonas aeruginosa* was inhibited by the methanolic extract of *Buchanania lanzana Spreng* [64]*.* The biofilms of foodborne pathogens, *S. typhimurium, S. aureus,* and* P. aeruginosa,* were reduced by 51.96%, 47.06%, and 45.28%, respectively, using the ethanol extract of *Amomum tsaoko* (*Zingiberaceae*) [65]. Garlic extract could inhibit the signaling system in *P. aeruginosa* infections and attenuate their biofilm formation [66]. Similarly, water-soluble cranberry extracts inhibited the biofilm formation of *V. cholerae* [67]. It also increased susceptibility toward tobramycin and azithromycin antibiotics and toward phagocytosis mediated by neutrophils [68].

### Targeting histidine kinase receptors in Gram-positive bacteria

**Fig. 5:**
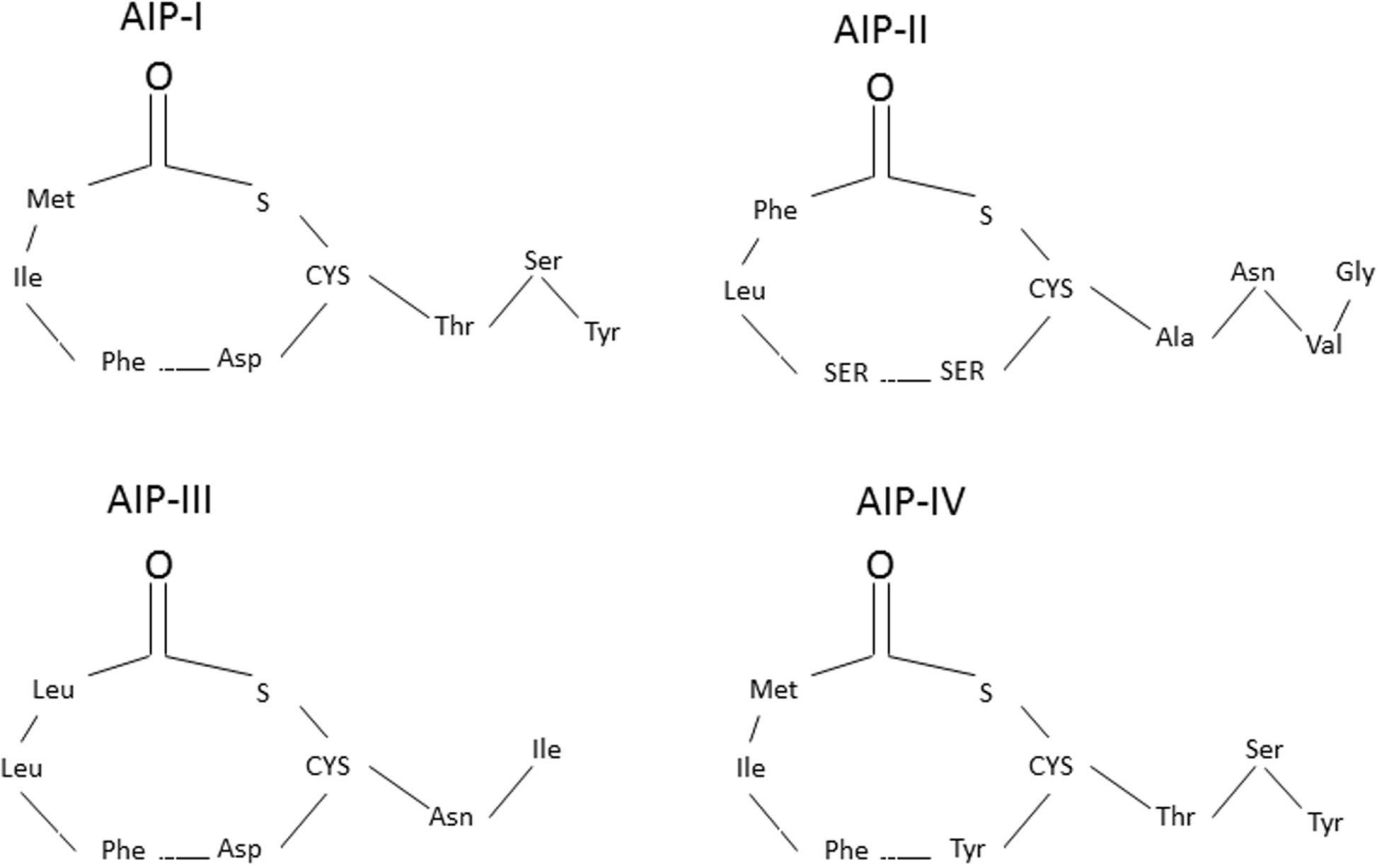
Chemical structure of autoinducer peptides (AIPs) in *S. aureus*

Two QS systems are regulated in Gram-positive bacteria a reactive transcriptional regulator and a histidine kinase receptor attached to the membrane [69]. Now, targeting these receptors with specific AIP antagonists can inhibit the action of the Gram-positive bacterial receptor and pathogenesis [41]. For example, the *S. aureus* agr system, which is an AIP-mediated QS system, uses four distinct thiolactone peptides (AIP I–IV) to control bacterial behavior [12, 44] (Fig. 5).

### Targeting LuxP receptors

The signaling of AI-2 is mediated by three protein receptors that have been described so far. The first is the LuxP gene in *V. harveyi*, the second is the LsrB gene in *S. typhimurium*, and the third is RbsB in *Aggregatibacter actinomycetemcomitans* [70]. The emergence of compounds capable of targeting these receptors is a promising strategy for silencing processes mediated by the AI-2 QS system [41, 71]. For instance, the sulphone compound showed an antagonistic effect on* V. harveyi* LuxP receptors [72]. Similarly, bioluminescence production by *V. harveyi* was inhibited by certain aromatic groups, such as polyols and phenylboronics, at sub-MIC concentrations [73]. Furthermore, the nucleoside analog LMC-21 decreased biofilm formation in *V. cholerae*, *V. vulnificus*, and *V. anguillarum* and reduced pigment and protease production in *V. anguillarum* [74].

### Enzymatic inactivation of auto-inducers

Enzymatic inactivation or degradation of extracellular antimicrobial peptides is a promising strategy because it can significantly reduce microbial resistance without placing stress on the bacterial cell. Certain enzymes, such as lactonase and acylase, can degrade AHL in Gram-negative bacteria by inactivating the lactone ring [75]. Oxidoreductases are a different class of enzymes that may interfere with bacterial communication [76] (Fig. 4C).

### Lactonase enzyme

**Fig. 6:**
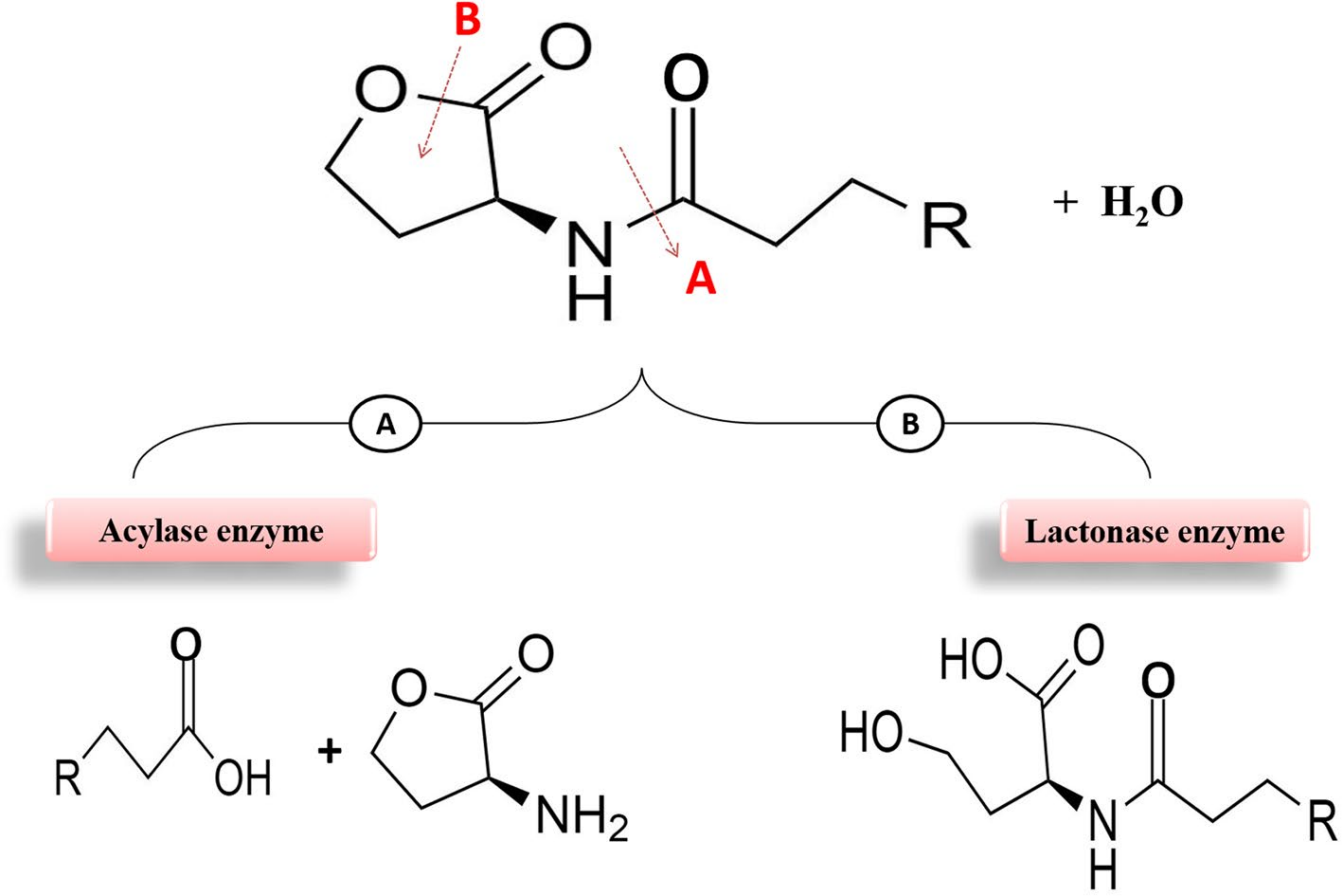
Enzymatic inactivation action of auto-inducers by acylase enzyme (**A**) and lactonase enzyme (**B**)

The lactonase enzyme acts by hydrolyzing the ester bond of the homoserine lactone ring (Fig. 6A), and the first microorganism to produce the lactonase enzyme was the *Bacillus* species that were isolated from soil [77]. Similarly, *Agrobacterium tumefaciens* [78, 79], *Rhizobium* sp. [80], *Chryseobacterium* sp. [81], *Mycobacterium avium* [82], *Microbacterium testaceum* [83]*, V. cholerae* [84],* Brucella melitensis* [85]*, Arthrobacter sp*. IBN110 and *Klebsiella pneumonia* [86]. These enzymes degrade all signals and have the broadest spectrum of AHL specificity regardless of acyl-side chain length or substitutions [87].

The lactonase enzyme has also been identified in mammalian tissues in the form of paraoxonases (PONs), which belong to the three categories PON1, PON2, and PON3. PON1 and PON3 genes have been detected in the kidneys and liver, and their amino acid products are detected in the blood circulation associated with high-density lipoprotein (HDL). In contrast, PON2 showed the best lactonase activity and was present in a variety of tissues [88].

### Acylase enzyme

The acylase enzyme breaks down the amide bond between the fatty acid side chain and lactone ring (Fig. 6B), as it can use AHL molecules as the sole energy source and nitrogen [89]. *Variovorax paradoxus* is the first microorganism reported to produce the acylase enzyme. Similarly, the acylase enzyme was produced by *Ralstonia* strain XJ12B [90], *Pseudomonas* strain PAI-A, *P. aeruginosa* PAO1 [91], and the kidney in Porcine [92]. The acylase enzyme has more substrate selectivity than lactonase as it can identify the acyl chain, such as the acylase PvdQ of *Pseudomonas* [93].

### Oxidoreductase enzyme

The oxidoreductase enzyme reduces or oxidizes the acyl chain of AHLs rather than breaking them down, as in acylase and lactonase enzymes. This inhibits AHL receptor binding and further gene expression controlled by QS [76]**.** For instance, the oxidoreductase enzyme produced by *Burkholderia* sp. GG4 inhibited the virulence factors of *Erwinia carotovora* by the modification of the 3-oxo-C6-HSL signaling molecule [94].

### Antibodies

It has also been reported that antibodies play a role in the inhibition of QS signaling molecules. [33]. Some organisms have receptors that inhibit QS compounds, and adaptive mammalian immune systems produce antibodies in response to antigen exposure. Signals from artificial intelligence are not expected to stimulate the human immune system because they lack protein and have a low molecular weight. However, it has been found that AHL bacterial molecules act as small molecule toxins in mammalian cells, inducing apoptosis and modulating NF_-k_B activity [33, 95]. For instance, the XYD-11G2 antibody prevented *P. aeruginosa* from producing pyocyanin and neutralized the 3-oxo-C12-HSL signal [96]*.* Monoclonal antibodies have also been shown to neutralize AIPs and disrupt QS pathways, though less attention has been paid to them. The S. aureus agr pathogen and E. faecalis agr-like systems were studied as AIP-mediated QS systems in Gram-positive bacteria [97]. For example, the AP4-24 H11 antibody could act as a sequester of the AIP-4 produced by S. aureus and inhibit QS pathogenicity in vivo and in vitro [98].

### Active uptake of AI signaling molecules by beneficial bacteria

As competing bacteria, certain other bacteria, such as those in the family Enterobacteriaceae, can sequester and disrupt cell communication. These pathogens include *B. anthracis*, *E. coli* O157, commensal *E. coli* K12, *Salmonella typhimurium*, and *Salmonella meliloti* [99]. Other members will no longer be able to use AI-2 signals to control their behavior because it takes AI-2 out of the environment. It was reported that when *E. coli* was cultured with *V. harveyi*, the bioluminescence mediated by QS signals was decreased by 18%. In contrast, using a mutant *E. coli* strain that contains a constitutively inhibited LsrK and decreased the bioluminescence by 90% [99], as the mutant *E.coli* disrupted the QS signaling system of *V. harveyi.*

### Application of molecularly imprinted polymers

The application of molecularly imprinted polymers has been proposed as an innovative approach. The authors have developed various polymers based on computational modeling to have the ability to form complex compounds that act as sequesters for the signals and eliminate the extracellular signals from the environment. The first generation of these polymers could target the signals of *V. fischeri* and inhibit biofilm formation and bioluminescence production. Some other polymers were designed to target AHLs signals in *P. aeruginosa* and inhibited 80% of QS-regulated biofilm [100]*.* For instance, the application of salicylate-based polymers exhibited slower adhesion rates, and the development of *P. aeruginosa* biofilms was significantly decreased compared to that of a control polymer [101]

### Targeting the efflux pumps

Efflux pumps play a significant role in the MDR of some antimicrobial agents as they have transport proteins that prevent these antimicrobial agents from reaching the target sites [102]. Recent research has demonstrated that QS is required for the development of efflux pumps in bacteria [103, 104]. For instance, it was found that *P. aeruginosa’s* active efflux pump and QS were related, and the addition of the C4-HSL signaling molecule enhanced the expression of the MexABOprM efflux pump in *P. aeruginosa* [105, 106]. So, inhibiting QS mechanisms play a significant role in the MDR of some antimicrobial agents [102]. Additionally, some substances such as phenothiazines and trifluoromethyl ketones (TFs) prevented bacteria’s QS systems from functioning properly, resulting in reduced production of efflux pumps and QS-regulated virulence factors [107]. Similarly, TFs approved efficacy in the inhibition of QS and efflux pump of *E. coli* and *C. violaceum* CV026 [108]. Moreover, phenylalanine arginyl b-naphthylamide efflux pump inhibitor significantly eliminated the virulence factors of *P. aeruginosa* as protease, elastase, pyocyanin, and motility and suppressed the QS cascade [109].

### Applications of QSI in different fields

#### Health

The human body and bacteria are closely associated with each other. Recent studies have shown that the human body has 10 trillion bacterial cells on the skin, known for being the largest organ as it is full of bacteria and dominated by *Staphylococcus*, *Proteobacteria*, *Corynebacteria*, and *Propionibacterium* [110]*.* The gut contains approximately 160 species of bacteria [111] that contribute to the human body's health and fitness, especially those belonging to *Firmicutes* and *Bacteroidetes* [112]. The body appears healthy when Firmicutes and Bacteroidetes make up 44% to 48% of the gut. However, when more carbohydrates are consumed than the body requires, the Bacteroidetes grow on these carbohydrates, causing obesity because they make up 82% to 86% of humans [113]. *Firmicutes* and *Bacteroidetes* work silently in our bodies; we do not always feel them in silence or feel it. However, another group of bacteria forces our body to listen because they are pathogenic and cause many diseases and symptoms that can lead to death if it is not controlled, and in some cases, antibiotic resistance [114]. QS signals are the key regulator of pathogenicity and virulence factors. Consequently, controlling these signals with the aforementioned strategies will significantly impact bacterial resistance and human health [115–117] by targeting the efflux pump gene expression and inhibiting biofilm formation. Conversely, these signals inhibited human breast cancer cell lines [118]. Therefore, the application of genetically modified bacteria with QS signals is an effective method for targeting cancerous cells (Choudhary and Dannert 2010). The use of QSI-coated plastics and polyurethanes on medical implants and catheters can be a practical strategy for medical applications. For example, dispersin B could inhibit *Staphylococcus epidermidis* from forming a biofilm. A combination of dispersin B with certain antibiotics such as triclosan demonstrated a synergistic effect and inhibited the formation of QS and biofilms. of *E. coli, S. aureus*, and *S. epidermidis* ( [119, 120]*.*

### Agriculture

It is well known that bacteria and plants have mutually beneficial relationships. For instance, epiphytes such as *Pantoea*, *Pseudomonas*, and *Erwinia* support *Nicotiana* (tobacco) plants by influencing the behavior of plant pathogens and assisting the host in triggering its defense mechanisms against pathogenic attacks [121]. Furthermore, the expression of the lactonase enzyme that inhibits AHL may protect transgenic plants like potatoes, cabbage, and tobacco from infection caused by *Erwinia carotovora*. The application of these inhibiting enzymes is effective in plant protection and controlling the virulence factors and pathogenesis of infectious organisms [83]. The release of certain chemical compounds during bacterial infection is another method to protect some plants, such as *Pisum sativum* and *Medicago truncatula*, and increase crop yield from bacterial infection. These compounds act as QS mimics, significantly controlling bacterial pathogens' virulence factors [41].

### Water treatment

Recent studies have demonstrated that biofilm is associated with the vast majority of bacterial infections in humans [122]. Biofilm development is crucial in treating bacterial infections and MDR because it exhibits new biological characteristics and enhanced environmental adaptability [123, 124]. Furthermore, bioreactors used for desalination and reclamation of seawater as well as on any artificial objects submerged in marine water, cause biofouling phenomena [125]. This phenomenon begins with the formation of biofilm, which is composed of microorganisms and microalgae, followed by the settlement of invertebrate larvae and algal spores. This has serious implications and increases the costs of fuel shipping and treatment [126]. The most prevalent bacteria are *Pseudomonas putida* and* Aeromonas* [127]*.* There are several strategies to control biofouling, (*i*) physical methods such as surface modification, but their application on a wide scale is not easy as more studies are needed to understand the adhesion mechanisms of organisms. (*ii*) Chemical methods, such as the use of antifouling paints such as tributyltin, are very toxic for marine organisms [128]. (*iii*) The application of biological strategies involves eco-friendly systems such as coatings with QSIs, using genetically modified marine organisms, or immobilizing QQ enzymes. [129]. Due to the short lifespan of enzymes, this highly effective method cannot be implemented on a large scale [130, 131]. Using a filtration membrane coated with the acylase enzyme, for instance, inhibited biofilm formation, exopolysaccharide production, and an anti-biofouling activity [130].

### Aquaculture and fisheries

Aquaculture and fisheries departments are a highly viable food source, especially for those living in coastal regions [132]. Nevertheless, pathogenic bacteria cause significant harm to aquatic organisms and substantial economic loss. The application of antibiotics to protect aquatic organisms is a simple option, but it can lead to MDR, particularly when they are used in sub-MIC concentrations [1]. Consequently, QSIs must be utilized to control the pathogenicity and virulence factors of these pathogens [133]. Some micro- and macroalgae produce QSIs, like *Chlorella saccharophila* CCAP211, which decreases the bioluminescence of *V. harveyi* and reduces the violacein production in *C. violaceum* CV026 without any effect on growth [134]. Moreover, *Chlamydomonas reinhardtii* microalga inhibited some QS-mediated phenotypes, such as luminescence [135]. Similarly, the red macroalga *Delisea pulchra* was reported to produce halogenated furanone that inhibited QS-regulated phenotypes [136]. Some sponges, such as *Haliclona megastoma*, *Aphrocallistes bocagei*, and *Clathria atrasanguinea* approved for their efficacy against violacein pigment production in *C. violaceum* CV026 and ATCC 12,472. They also inhibited biofilm formation, protease, and hemolysin production of *S. marcescens* [137]*.*

### Development of resistance against QSIs

QS inhibition has been considered a potential alternative antipathogenic therapy. It has been demonstrated to be an efficient anti-infective technique in several host-microbe systems [45]. Prior studies reported that pathogens are unlikely to develop resistance to the QSI tactics because they pose no selective pressure on bacteria [5, 12, 138]. However, some studies showed the possibility of development of resistance against QSI [139]. Due to nalC and mexR mutations, *P. aeruginosa* developed resistance to the QSI; brominated furanone C-30 [140]. Similarly, García-Contreras et al. [141] reported that *P. aeruginosa* developed resistance against 5-fluorouracil QSI in addition to brominated furanone C-30 for at least one QS-regulated phenotype.

## Conclusion

The emergence of resistance is the result of a natural evolutionary process that encourages the development of resistant strains through selection pressure. Furthermore, the formation of bacterial biofilms has been identified as one of the most challenging aspects of treating clinical bacterial infections. Additionally, the QS system has been shown to regulate bacterial efflux pumps and biofilm formation in multidrug-resistant bacteria. Consequently, QQ strategies could offer a novel approach to combating microbial resistance by regulating the expression of efflux pump genes and inhibiting bacterial invasiveness and infectiousness. Antimicrobial resistance regulation through control of QS signaling molecules in clinical isolates requires additional research. Consequently, innovation and advancement in research on QQ molecules and their effects on regulatory systems will result in the introduction of new QQ molecules to combat microbial resistance. The discovery of QQ molecules is expected to increase significantly over the upcoming decades, and they could be used as coating agents in medical devices, replacing bacteriostatic and bactericidal antibiotics in-vivo.

## Data Availability

The original contributions presented in the review are included in the article material. Further inquiries can be directed to the corresponding authors.
